# Intravesical real-time imaging and staging of bladder cancer: Use of optical coherence tomography

**Published:** 2008

**Authors:** Vengatesh K. Sengottayan, Pawan Vasudeva, Divakar Dalela

**Affiliations:** Department of Urology, C.S.M.M.U (Upgraded King George Medical College), Lucknow - 226 003, Uttar Pradesh, India

## SUMMARY

In this retrospective study, the authors have assessed the application of optical coherence tomography (OCT) as an adjunct to conventional cystoscopy in the endoscopic evaluation of bladder tumors. From December 2005 to April 2007, 32 patients (25 men and seven women with median age of 59 years), majority of whom were known or newly diagnosed cases of bladder cancer, underwent OCT in addition to conventional rigid cystoscopy by a single surgeon. The US FDA-approved Niris Imaging System was used to acquire OCT images. The images of normal and suspicious areas were obtained prior to biopsy/resection using a flexible fiber optic probe inserted through the working channel of the rigid cystoscope. OCT images were obtained at representative portions of the lesion as well as at the junction of normal-appearing urothelium by placing the end-viewing optical probe perpendicular to the tissue and holding it in contact with the tissue for approximately 1.5 sec. The OCT image findings were interpreted intraoperatively at the time of the scan. Scan findings were later compared with the final pathologic diagnosis which was obtained by resecting the area of interest and submitting the tissue in formalin.

Images of a total of 38 suspicious areas were correlated with biopsy findings. Among 20 lesions staged as Ta on biopsy, OCT accurately identified 18 whereas in two OCT overstaged the disease hence giving a sensitivity of 90% and specificity of 89% for Ta lesions. OCT was able to demonstrate invasion in 11 out of 11 patients who had either lamina propria or muscle invasion. Among four lesions invading lamina propria on biopsy, OCT correctly diagnosed three whereas one was staged as T2 giving a sensitivity and specificity of 75% and 97%, respectively. OCT accurately identified muscle invasion in seven of seven biopsy-proven muscle-invasive tumors giving a sensitivity and specificity of 100% and 90%, respectively. The negative predictive value of OCT for muscle invasion was 100%. In one patient Carcinoma *in situ* (CIS) on biopsy was correctly identified by OCT, while it could be ruled out in another by OCT. OCT was able to differentiate malignant from benign lesions with a positive predictive value of 89% and negative predictive value of 100%.

Based on the above findings, the authors concluded that OCT is a rapid, real-time, high-resolution, easy-to-use tool that can help differentiate Ta and T1 tumors and identify muscle-invasive bladder tumors.

## COMMENTS

Conventionally, white light cystoscopy in combination with transurethral resection is used for assessing depth of tumor penetration.[1] Unfortunately, the frequency of tumor understaging is relatively high. In one study, 40% of patients with non-muscle-invasive clinical stage had muscle-invasive disease as confirmed by the final pathological report obtained after radical cystectomy.[2] Other imaging modalities including computed tomography (CT), magnetic resonance imaging (MRI), and ultrasound have limited ability in resolving the microstructural detail required to assess subtle changes in the bladder wall.

OCT, a technology already in use in the diagnosis of ophthalmological diseases, has been investigated as a tool for staging bladder cancer in this study. It is analogous to ultrasound B mode imaging except that it uses near infrared light as opposed to sound waves.[3] With a resolution of 10 to 20 μm, it is akin to using an optical microscope and micro-architectural features of the bladder wall can be seen.[4] The images obtained are cross-sectional and in real-time. As the depth of penetration is only 1 to 2 mm, though it is able to distinguish structural changes in the bladder wall involving the mucosa, lamina propria, and superficial muscularis properly, information on invasion of deeper layers and extravesical spread is not available.[5] In the OCT image, the mucosa generally appears as a dark, thin, clearly defined layer. The lamina propria gives a bright, distinct signal, whereas muscularis shows a darker, spindled appearance.[1]

Key benefits of OCT are live sub-surface images at near-microscopic resolution, without the hazard of ionizing radiation and without the need for any preparation of the sample.

In the future OCT may find an application in the early diagnosis and screening of mucosal malignancies. It may also have a potential role in confirming completeness of resection after transurethral resection of bladder tumour (TURBT) once images obtained from tissues affected by heat are standardized.
